# Supplementary material for: Effects of a Combined Method of Normobaric Hypoxia on the Repeated Sprint Ability Performance of a Nine-Time World Champion Triathlete: A Case Report
Source: Behav Sci (Basel). 2024 Nov 12;14(11):1084. doi: 10.3390/bs14111084 (PMC11591127; doi:10.3390/bs14111084)
Supplement: Supplementary file 1 [file behavsci-14-01084-s001.zip › behavsci-3191194-supplementary.pdf]

CARE case report guidelines

CARE Checklist of information to include when writing a case report

(cc) BY-NC-ND

Topic	Item	Checklist item description	Reported on Line
Title	1	The diagnosis or intervention of primary focus followed by the words "case report"	1
Key Words	2	2 to 5 key words that identify diagnoses or interventions in this case report, including "case report"	23
Abstract (no references)	3a	Introduction: What is unique about this case and what does it add to the scientific literature?	10-11
	3b	Main symptoms and/or important clinical findings	13-16
	3c	The main diagnoses, therapeutic interventions, and outcomes	19-21
	3d	Conclusion—What is the main "take-away" lesson(s) from this case?	21-22
Introduction	4	One or two paragraphs summarizing why this case is unique (**may include references**)	27-30
Patient Information	5a	De-identified patient specific information	52-54
	5b	Primary concerns and symptoms of the patient	49-54
	5c	Medical, family, and psycho-social history including relevant genetic information	49-54
	5d	Relevant past interventions with outcomes	56-57
Clinical Findings	6	Describe significant physical examination (PE) and important clinical findings	56-69
Timeline	7	Historical and current information from this episode of care organized as a timeline	70
Diagnostic Assessment	8a	Diagnostic testing (such as PE, laboratory testing, imaging, surveys).	75-115
	8b	Diagnostic challenges (such as access to testing, financial, or cultural)	56-58
	8c	Diagnosis (including other diagnoses considered)	49-54
	8d	Prognosis (such as staging in oncology) where applicable	46-47
Therapeutic Intervention	9a	Types of therapeutic intervention (such as pharmacologic, surgical, preventive, self-care)	56-70
	9b	Administration of therapeutic intervention (such as dosage, strength, duration)	70
	9c	Changes in therapeutic intervention (with rationale)	69
Follow-up and Outcomes	10a	Clinician and patient-assessed outcomes (if available)	117-151
	10b	Important follow-up diagnostic and other test results	117-151
	10c	Intervention adherence and tolerability (How was this assessed?)	117-151
	10d	Adverse and unanticipated events	70-71
Discussion	11a	A scientific discussion of the strengths AND limitations associated with this case report	153-210
	11b	Discussion of the relevant medical literature **with references**	156-158;166-168;189-191
	11c	The scientific rationale for any conclusions (including assessment of possible causes)	189-191
	11d	The primary "take-away" lessons of this case report (without references) in a one paragraph conclusion	202-210
Patient Perspective	12	The patient should share their perspective in one to two paragraphs on the treatment(s) they received	X
Informed Consent	13	Did the patient give informed consent? Please provide if requested	Yes ☒ No ☐
